# Wafer-Scale Fabrication of Wearable All-Carbon Nanotube Photodetector Arrays

**DOI:** 10.1021/acsnano.4c01087

**Published:** 2024-07-12

**Authors:** Peng Liu, Er-Xiong Ding, Zhenyu Xu, Xiaoqi Cui, Mingde Du, Weijun Zeng, Anastasios Karakassides, Jin Zhang, Qiang Zhang, Faisal Ahmed, Hua Jiang, Pertti Hakonen, Harri Lipsanen, Zhipei Sun, Esko I. Kauppinen

**Affiliations:** † Department of Applied Physics, Aalto University, Espoo FI-00076, Finland; ‡ Department of Electronics and Nanoengineering, Aalto University, Espoo FI-00076, Finland; § QTF Centre of Excellence, Department of Applied Physics, Aalto University, Espoo FI-00076, Finland

**Keywords:** lithography-free, chemical-free, wearable electronics, all-carbon nanotube devices, photodetector

## Abstract

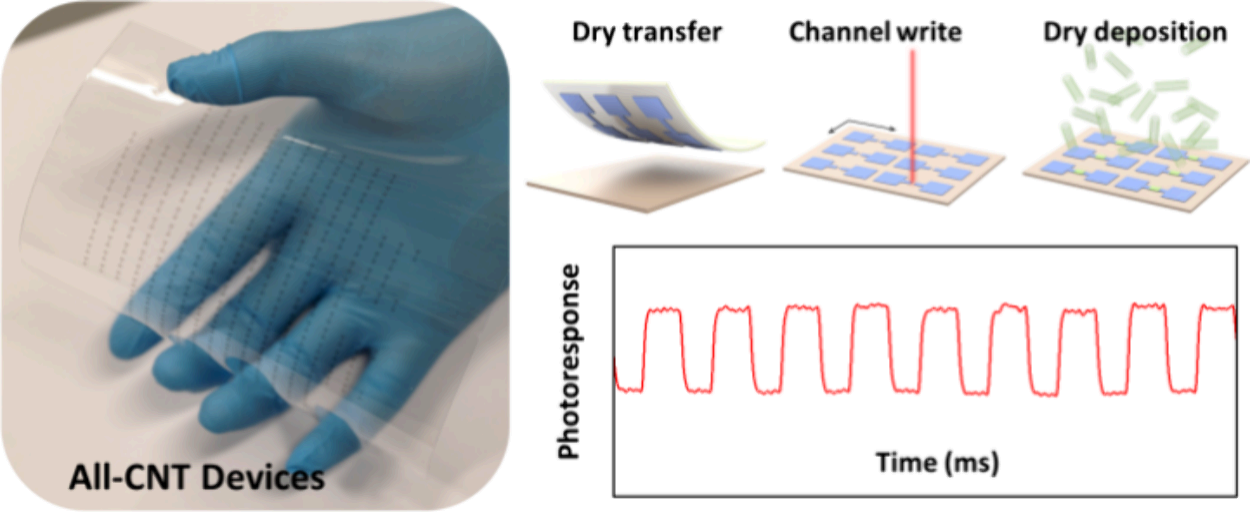

With electronic devices evolving toward portable and high-performance wearables, the constraints of complex and wet processing technologies become apparent. This study presents a scalable photolithography/chemical-free method for crafting wearable all-carbon nanotube (CNT) photodetector device arrays. Laser-assisted patterning and dry deposition techniques directly assemble gas-phase CNTs into flexible devices without any lithography or lift-off processes. The resulting wafer-scale all-CNT photodetector arrays showcase excellent uniformity, wearability, environmental stability, and notable broadband photoresponse, boasting a high responsivity of 44 AW^–1^ and a simultaneous detectivity of 1.9 × 10^9^ Jones. This research provides an efficient, versatile, and scalable strategy for manufacturing wearable all-CNT device arrays, allowing widespread adoption in wearable optoelectronics and multifunctional sensors.

The landscape of traditional electronics is evolving from bulky solid-state to portable, high-performance, multifunction, and flexible/wearable devices.^1,2^ Significant progress has been achieved in the materials, designs, and manufacturing processes for wearable system subcomponents, including transistors, sensors, diodes, energy storage components, and so on.^3−5^ Carbon nanotube (CNT) is an ideal candidate for flexible electronics due to their quasi-one-dimensional topology and outstanding properties, such as high intrinsic carrier mobility, electrical conductivity, mechanical flexibility, good chemical stability, and the potential for low-cost as well as wafer-scale production.^6−8^ To date, numerous studies have exploited the high conductivity of CNTs to substitute indium tin oxide (ITO) or traditional noble metal electrodes such as gold and palladium in flexible devices.^9−12^ Zhang et al.^10^ realized that high-performance ITO-free perovskite solar cells using CNT films significantly enhanced mechanical robustness and environmental stability. Jang et al.^12^ presented the strong dark current suppression in flexible organic photodetectors by CNT electrodes, realizing high detectivity. In addition, utilizing the semiconducting characteristics of CNTs to replace the traditional semiconductor in the channel of flexible devices has also been widely studied.^13,14^ Overall, CNTs have shown great potential in creating flexible electronics and transforming various aspects of our future lives. However, the research on CNTs in flexible electronics is still in the stage of partial component substitution. The research on all-carbon nanotube (all-CNT) devices constructed entirely from CNTs remains relatively scarce, especially in the in-depth investigation and wafer-scale fabrication of flexible all-CNT devices.^4,5,15−17^ For instance, in 2006, Cao et al.^15^ developed the highly bendable thin film transistors using CNT-based conductors and semiconductors via layer-by-layer transfer printing. The fabrication began with applying a photocurable epoxy adhesive layer for CNT film transfer onto a flexible substrate using an elastomeric stamp, forming the gate electrode. Subsequently, an insulating aluminum oxide layer was deposited and treated with oxygen (O_2_) plasma. Semiconducting single-walled CNTs (s-SWCNTs) were similarly transferred to the dielectric layer. The CNTs of the source and drain electrodes were patterned on the silicon substrate through photolithography and O_2_ plasma etching. The final steps involve conducting photolithography and O_2_ plasma etching to achieve electrical isolation between devices. Zou et al.^16^ used three transfer methods combined with two times of lithography and O_2_ plasma etching technology to fabricate all-CNT transistors on a flexible substrate.

While this progress has been made, they predominantly relied on traditional fabrication technologies involving complex processes and wet chemical processing. This limits the diversity of active materials and flexible substrates. In general, lithography, performed on rigid substrates like silicon wafers, requires an extra lift-off step to write patterns onto flexible substrates, especially for the substrates with poor heat resistance, such as polydimethylsiloxane (PDMS), poly(methyl methacrylate) (PMMA), and hydrogels.^5,18^ In addition, the inevitable chemical contamination resulting from the lift-off process, which includes concerns such as photoresist residue and solvent-induced damage on the substrate and material, leads to the degradation in device performance.^19,20^ In addition, lithography equipment and processes demand high investment and operating costs, preventing wearable devices from spreading and popularizing. Simplifying the manufacturing processes and reducing fabrication costs of wearable devices are also critical issues in achieving commercial scale.^5^ Recently, printing technology^21^ has been widely adopted due to its simple process and low substrate restrictions. However, several issues still need to be addressed, such as structural damage of CNTs in the solution treatment, chemical contamination from the dispersant and surfactant, and low CNT separation efficiency.^8^ Additionally, achieving wafer-scale highly conductive electrodes with a practical thickness requires a long printing time. Until now, the technologies mentioned above have difficulties in meeting the growing product demand and commercial development, and there is an urgent need to develop a simple and versatile method for the large-scale manufacturing of wearable electronic devices.

This work demonstrates a scalable photolithography-free dry method to fabricate wearable all-CNT photodetector device arrays. Specifically, laser-assisted patterning and dry deposition techniques were used to directly assemble gas-phase CNTs into device arrays. Double-walled CNTs (DWCNTs) with high conductivity are used as flexible electrodes, and highly enriched s-SWCNTs serve as the channel material. The wafer-scale all-CNT device arrays exhibit excellent uniformity, multifunctionality, ohmic contact characteristics, wearability, transparency, and environmental stability. Furthermore, the wearable photodetector exhibits a high responsivity of 44 AW^–1^ and a detectivity of 1.9 × 10^9^ Jones in the visible region. An exceptional responsivity of 2 AW^–1^ at telecommunication wavelength has been achieved. Furthermore, beyond photodetectors, the methodology presented here can be applied to fabricate various electronic devices, such as wearable sensors and transistors.

## Results and Discussion

**Figure 1:**
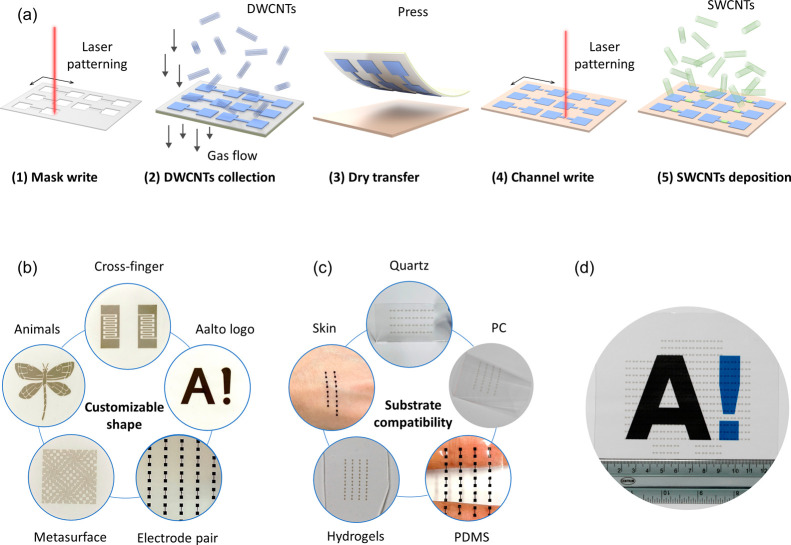
Schematics, flexibility, and scalability of laser-assisted CNT patterning and dry deposition. (a) Schematics of the preparation of all-CNT devices. The sizes of the components in the schematics are not actual proportions. In step 5, SWCNTs are randomly deposited on the substrate surface under a temperature gradient. The schematic shows the SWCNTs in the channel, which is in an electrical connection state. The remaining areas also have SWCNTs, but these devices are electrically isolated from nearby devices. (b) CNT patterns with customizable shapes on the membrane filter. (c) CNT patterns are transferred to various substrates. (d) Array of wafer-scale all-CNT devices with channels and contacts.

Figure 1a illustrates the fabrication process of the wafer-scale all-CNT device array. It includes five steps. The first step is laser write, which uses a femtosecond laser to write customized patterns on polyethylene terephthalate (PET) film. Then, the PET pattern and MF-Millipore membrane filter were assembled through a laminator. In the second step, the patterned membrane filter was installed at a floating catalyst chemical vapor deposition (FCCVD) reactor outlet to collect CNTs. The CNTs are directly deposited on the membrane filter by gas flow to form various patterns, and the CNT thickness can be precisely controlled through collection time.^7^Figure 1b shows the diversity of custom patterns such as crossed electrodes, logos, electrode pairs, metasurface, and animal patterns. The third step is to transfer the patterned CNT film to the target substrate via dry pressing. It is worth noting that the entire transfer process can be completed within 1 min.^22^ Additionally, the patterned membrane filter can be reused multiple times after the transfer. This provides a potential path for batch fabrication of electrodes for wearable devices. Figure 1c shows the transfer of CNT patterns onto various substrates, e.g., quartz, polycarbonate (PC), PDMS, hydrogels,^23^ and skin, by dry pressing. With customized patterns, multisubstrate compatibility, and efficient dry transfer methods, we can prepare flexible electrodes and patterns that meet the needs of various scenarios. This avoids the conventional tedious photolithography and complex wet transfer processes. In addition, dry transfer allows the material to be protected from chemical contamination and retain its original properties.^7,22^ Up to now, CNT flexible electrodes have been available for use in devices, but the channel length is long (>100 μm), which is not conducive to the miniaturization of devices. Therefore, the fourth step is to write micrometer-scale channels directly in the CNT pattern on the substrate, making it possible to prepare miniaturized devices. Four channel lengths below 100 μm are discussed later, with the shortest reaching 15 μm, which is limited by the laser resolution. Finally, the substrate with a CNT pattern was placed in a cavity with a temperature gradient, and the SWCNTs synthesized by FCCVD were then introduced into the cavity through the airflow. Under the thermophoresis effect, the SWCNTs are randomly and uniformly deposited on the substrate surface.^24^ The SWCNT density can also be controlled through collection time and temperature gradient to meet the needs of multifunctional devices. The fabrication time of an entire array can be efficiently completed within 40 min to 2 h. More processing details, including optical and electronic images, are provided in Figure S1. Additionally, we fabricated a flexible device array with a 4 in. size, as displayed in Figure 1d. Through electrical characterization, we found that the arrays exhibit high electrical uniformity (Figure S2).

**Figure 2:**
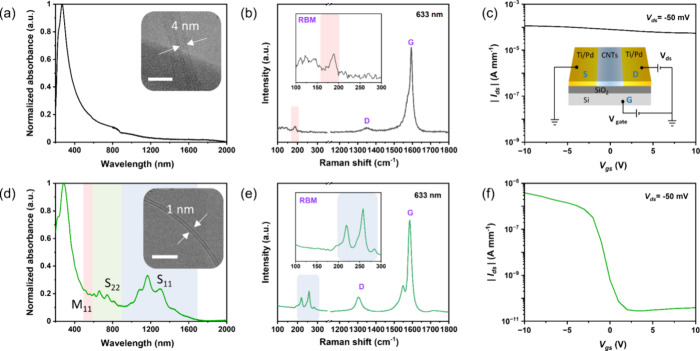
Material characterization. (a–c) Optical absorption spectrum, Raman spectrum, and the transfer curve of electrode material, respectively. The inset in (a) presents a TEM image illustrating the double-walled microstructure of the electrode material; the scale bar is 10 nm. The inset in (b) shows the radial breathing mode (RBM) peak of electrode material, with the colored region representing the characteristic peaks of metallic CNTs. The inset in (c) shows a schematic of the CNT transistor. (d–f) Optical absorption spectrum, Raman spectrum, and the transfer curve of channel material, respectively. The inset in (d) is the TEM image of the channel material, and the scale bar is 10 nm. The inset in (e) shows the RBM peak of channel material, with the colored region representing the characteristic peaks of semiconducting CNTs. The drain-to-source current and the channel width were utilized to determine the width-normalized current in (c) and (f).

The electrode and channel materials of wearable devices are composed of CNTs synthesized through the FCCVD method.^7,25^Figure 2a shows the ultraviolet–visible-near-infrared (UV–vis-NIR) absorption spectrum of the electrode material with the absence of van Hove characteristic peaks (S_11_, S_22_, and M_11_) of SWCNTs in the 200–2000 nm region, indicating a low ratio of SWCNTs in the electrode. The microstructure of the electrode material was further examined using a double Cs-corrected transmission electron microscope (TEM). A typical TEM image of the CNT is displayed in the inset of Figure 2a, revealing a distinct double-walled structure with a tube diameter of about 4 nm. Moreover, the Raman spectrum (Figure 2b) shows that our as-synthesized CNT electrode material is of high quality (high G/D peak ratio) and displays a strong peak near 180 cm^–1^, which corresponds to the metallic inner tube of DWCNTs.^26^ To understand the transfer properties of CNTs, bottom-gate transistors were fabricated using Ti/Pd as electrodes and as-synthesized CNTs as the channel materials.^7^Figure 2c presents the transfer curve of the CNT electrode material under a bias of −50 mV and gate voltage ranges of −10 to +10 V, and the flat curve indicates that the electrode material is metallic and can be selected as a conductive electrode for flexible devices. Additionally, through atomic force microscope (AFM) testing, it was determined that the thickness of the DWCNT electrode is approximately 40 nm (Figure S3). This thickness is comparable to conventional metal electrodes, indicating the potential for DWCNT electrodes to serve as an effective alternative in electronic device applications. In contrast, the absorption spectra of the channel material exhibit characteristic peaks of SWCNTs (Figure 2d). TEM characterization confirms that the channel material possesses a single-walled structure with a diameter of approximately 1 nm (inset of Figure 2d). Notably, the characteristic peaks S_11_ and S_22_, representing semiconducting tubes, are much stronger than the M_11_ peak representing metallic tubes, which indicates the dominance of s-SWCNTs in the channel. Specifically, the purity of s-SWCNTs in this work is about 94%, calculated using the method reported in our previous work.^7^ This observation is supported by the Raman spectrum (Figure 2e), where two strong peaks representing the s-SWCNTs appear in the Raman shift above 200 cm^–1^. Excitingly, the channel material exhibits an impressive On/Off ratio exceeding 10^5^ within a gate voltage range of −10 to +10 V, indicating its p-type semiconductor characteristics (Figure 2f). This property holds significant promise for the widespread application of s-SWCNTs in flexible optoelectronics and transistors.

**Figure 3:**
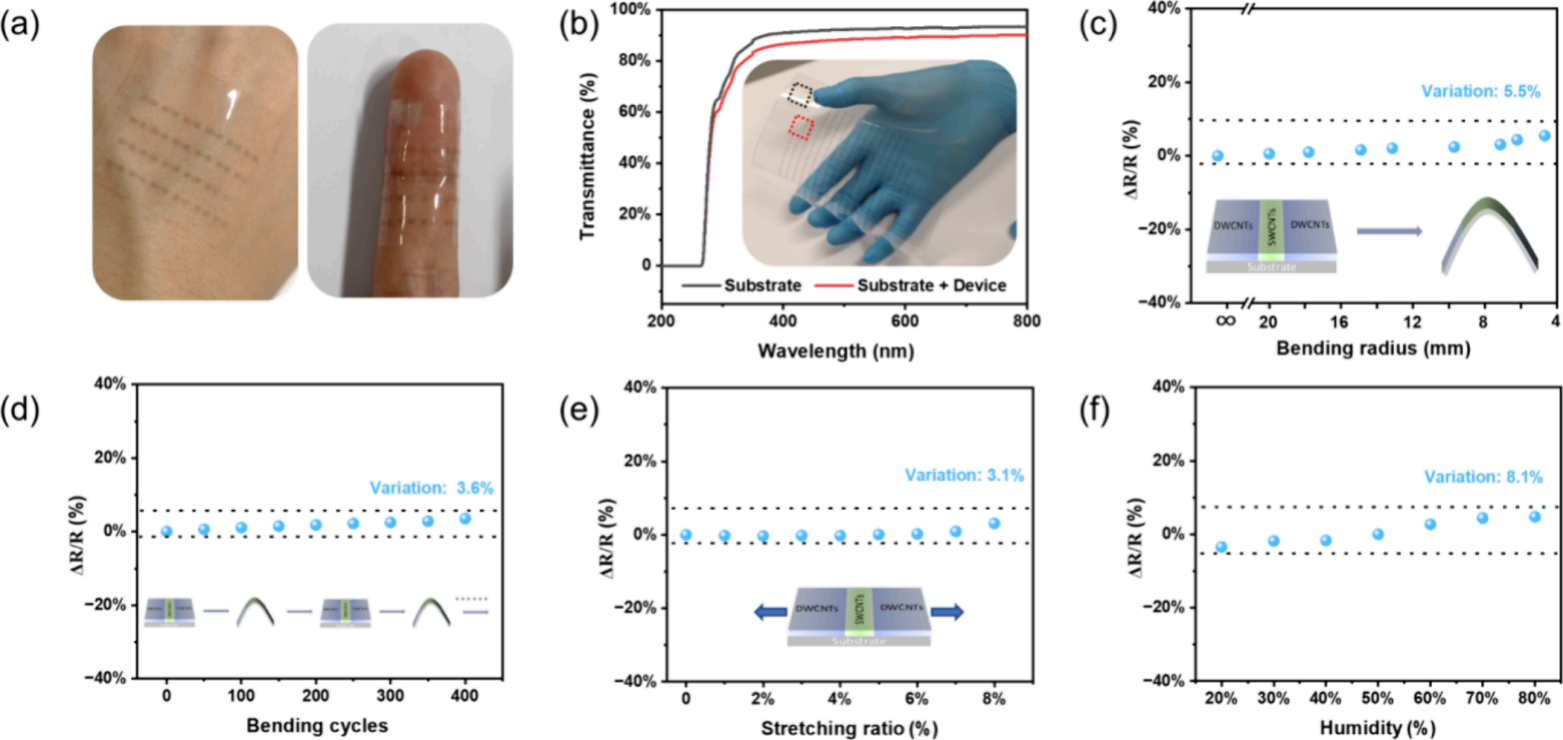
Transparency, wearability, and stability of all-CNT device arrays. (a) Optical images of all-CNT device arrays under normal and bending states. (b) Optical transmittance spectra of the PC and PC/all-CNT devices. (c) Relative resistance changes in all-CNT devices under various bending radii of 4.6–20 mm. (d) Relative resistance change measured over 400 bending cycles at a bending radius of 4.6 mm (corresponding to a strain of 2.6%). (e) Relative resistance changes in the all-CNT devices under various stretching ratios of 0–8%. (f) Stability of the all-CNT devices under different humidity environments. The stretching test of all-CNT devices was completed on the PDMS substrate, and other tests were completed on the PC substrate.

Then, we investigated the wearability and environmental stability of the all-CNT devices. Figure 3a displays an optical image of the fabricated all-CNT device array, affirming its components’ wearability and optical transparency. To quantitatively evaluate the device transmittance, UV–vis-NIR spectroscopy was employed across the wavelength range from 200 to 800 nm (Figure 3b). At a wavelength of 550 nm, the substrate and substrate/device transmittances are 92 and 88%, respectively. Excluding the bare substrate, the device absorption rate is around 4%. This high transmittance is attributed to the nanometer-scale of CNTs and precise control over the CNT film thickness, making our all-CNT device distinct from previous works using opaque metals as electrode materials.^8,15^ The stability of wearable devices is crucial for their functionality across diverse scenarios. To assess flexibility, we measured the resistance variation under various bending radii (Figure 3c). With a decreasing bending radius, the resistance experiences a marginal increase—only about 5.5% at a radius of 4.6 mm (equivalent to a strain of 2.6%).^5^ This change is significantly smaller than that observed in commonly used ITO transparent electrodes (>200% at a strain of 1.5%), fragile metal electrodes, and the CNT/graphene flexible device (∼7.4% at a radius of 5.5 mm).^4,5,27^ Durability testing involved cyclic bending at a radius of 4.6 mm (Figure 3d and Figure S4), which exhibits around 3.6% resistance variation over 400 cycles, underscoring the device’s outstanding electrical and mechanical stabilities. Furthermore, the resistance changes were examined under different stretching states (Figure 3e). A gradual increase in the stretching ratio results in a slight resistance increase, with a mere 3.1% change at an 8% stretching ratio. The flexibility and stretchability are superior to those of flexible devices made of quantum dots,^28^ nanowires,^29^ MXenes,^29^ or other two-dimensional materials.^30^ The exceptional mechanical properties stem from flexible and stretchable one-dimensional CNTs, ensuring a robust van der Waals contact within the interconnected network structure without compromising electrical connection. These characteristics effectively address the fragility challenges associated with rigid components in wearable devices.

In actual applications, devices are susceptible to various environmental factors, such as humidity, oxygen, and temperature. Here, we investigated how the device resistance responded to varying humidity environments. Notably, the device exhibited a modest 8.1% change in resistance as humidity ascended from 20% to 80% at room temperature (Figure 3f). The slight resistance increase is attributed to the hydrophobic nature of all CNT components. This inherent property distinguishes the all-CNT device, rendering it more robust in environmental stability than the devices utilizing air- and/or moisture-sensitive materials, such as perovskites.^31,32^ The all-CNT device array demonstrates commendable optical transparency, wearability, and environmental stability. These characteristics position it to meet the demanding requirements for employment in complex application scenarios.

**Figure 4:**
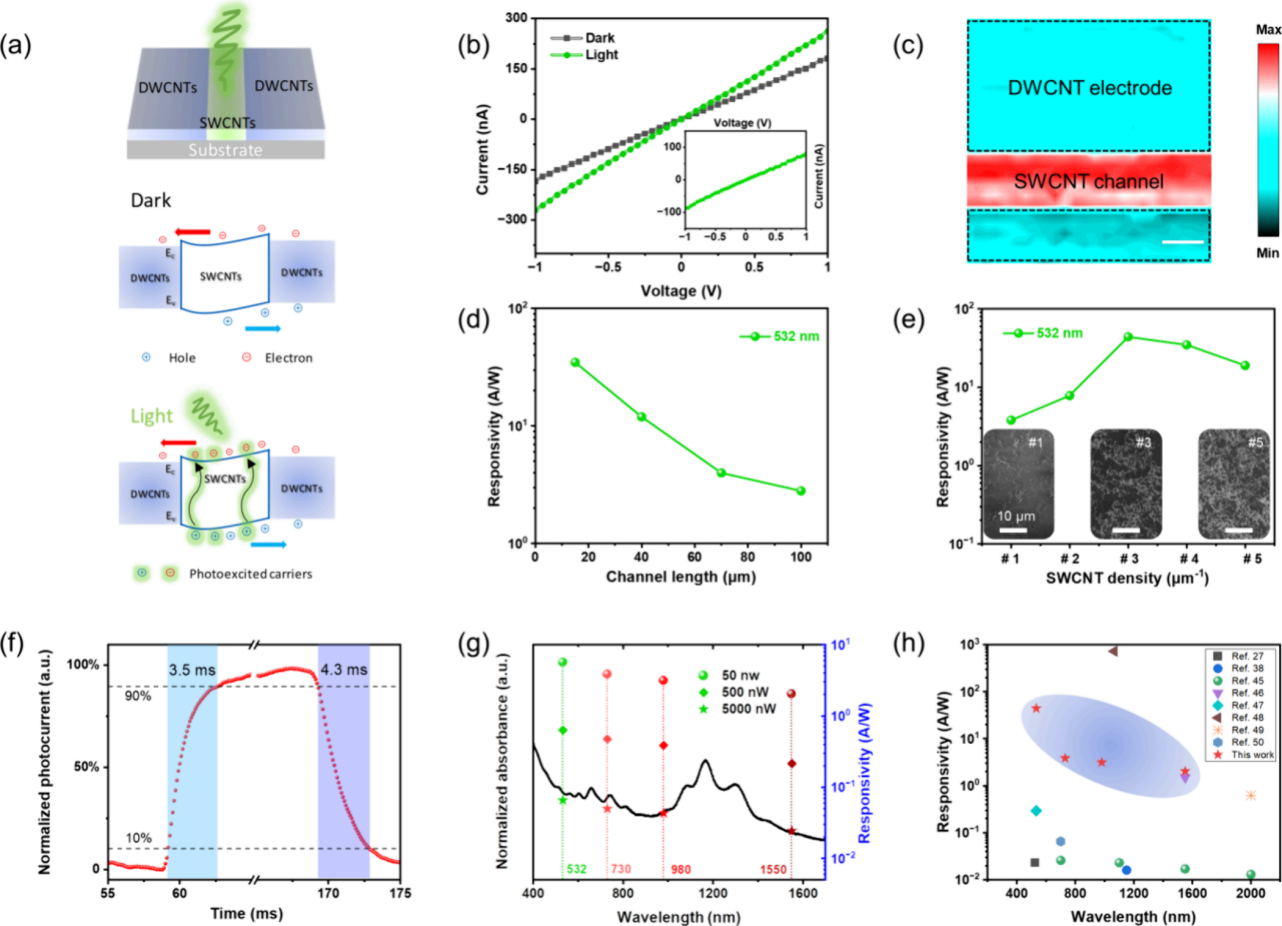
Optoelectronic properties of wearable all-CNT devices. (a) Illumination schematic and response mechanism of all-CNT photodetectors. (b) Plots of current against voltage curves under dark and 532 nm laser illumination environments and the inset plots are net photocurrent against voltage curves. (c) Photocurrent mapping. Laser power: 5 nW, 532 nm laser, scale bar: 10 μm, channel length: 15 μm, SWCNT density: #2. (d) Responsivity as a function of channel length. Laser power: 5 nW, SWCNT density: #4. (e) Responsivity as a function of SWCNT density. Laser power: 5 nW, channel length: 15 μm. The insets are SEM images of three different SWCNT densities, and the scale bar is 10 μm. (f) Response time of the photodetector, the rise time is 3.5 ms and the fall time is 4.3 ms. (g) Broadband photoresponse of all-CNT photodetector under four lasers of 532, 730, 980, and 1550 nm. (h) Comparison of the responsivities of our all-CNT photodetector with those of previously reported CNT-based photodetectors.

For practical applications of all-CNT devices, we investigate their optoelectronic properties. Initially, a focused continuous wave 532 nm laser was selected as the light source to conduct preliminary research on the photodetector (Figure 4a). Figure 4b displays the current–voltage (*I–V*) curves of the photodetector under dark (*I*_dark_) and illuminated (*I*_light_) conditions. The clear distinction of |*I*_light_| > |*I*_dark_| at a bias voltage of ±1 V indicates the positive response of our wearable all-CNT photodetector to visible light. A closer examination of the *I–V* curve reveals a linear increase in both the *I*_dark_ and the *I*_light_ with escalating voltage. This is attributed to the stronger electric field and lower energy barrier induced by elevated voltage, accelerating the carrier transport and increasing the currents accordingly. As illustrated in Figure 4a, photogenerated electron–hole pairs participate in charge transport upon laser illumination, and a higher external electric field facilitates their separation and transport, resulting in a swifter rise in *I*_light_.^33^ Additionally, the rectification ratio (forward and reverse current ratio) remains consistently around 1 under different bias voltages (Figure S5a). This steadfastness affirms the photoconductive nature of the fabricated photodetector, establishing an ohmic contact between the SWCNT channel and DWCNT electrodes. To further explore the origin of photocurrent, meticulous photocurrent mapping was conducted on the all-CNT photodetector under +1 V bias (Figure 4c). The result indicates that the primary photocurrent source is the s-SWCNTs in the channel rather than the DWCNTs in the electrodes.

Current responsivity (*R*_*λ*_), widely employed to evaluate photoelectric performance, quantifies the capability of photodetector to convert optical signals into electrical signals (the calculation method is in the Supporting Information).^11,34^Figure S5b displays the relationship between the responsivity and bias voltage. The responsivity of photodetector increases with bias voltage due to fast carrier extraction by the enhanced electric field, leading to a high photocurrent. All tests in this work were performed at a bias voltage of +1 V unless otherwise specified. Figure S6 shows the responsivity of photodetector under a 532 nm laser with different powers. Notably, the responsivity decreases with increasing incident light power due to the saturation of electron–hole pair generation and the reduction in carrier lifetime (τ_lifetime_) at a higher light power.^35^ Subsequently, the correlation between the performance of all-CNT photodetectors and their structural characteristics (channel lengths and SWCNT density) was investigated in depth. We found that the current of all-CNT photodetectors gradually decreases as the channel length increases (Figure S7a). This decline can be easily attributed to the elongation of the channel length, resulting in the formation of more CNT junctions and longer carriers’ transmission paths, thereby elevating the overall channel resistance. Meanwhile, many photoexcited carriers experience scattering, trapping, and recombination within the long channel before reaching the electrodes.^36^ Consequently, photodetectors with longer channels can only produce smaller photocurrents and worse photoresponse under identical illumination (see Figure 4d). Building upon the optimal channel length of 15 μm, we also controlled the number of CNTs in the channel by the thermophoresis method (refer to our previous report for details).^24^ According to the sample collection time, there are five samples with different CNT densities, denoted as #1, #2, #3, #4, and #5. Scanning electron microscope (SEM) images in Figure 4e depict samples #1, #3, and #5, showcasing a gradual increase in SWCNT density with prolonged sampling time, indicative of the highly controllable density of SWCNTs in the channel. The responsivity exhibits an interesting trend, initially increasing and then decreasing, as the SWCNT density rises. The initial augmentation in photodetector responsivity concerning SWCNT density can be ascribed to more SWCNTs, which absorb more photons and generate more photoexcited carriers. Simultaneously, the heightened CNT density provides additional transmission paths for carriers, facilitating their collection by electrodes (Figure S7b). Nonetheless, with a continued increase in SWCNT density, the probability of additional metallic SWCNTs interconnecting the channel rises, leading to increased dark current and moderate photocurrent. As is known, metallic SWCNTs have an ignorable contribution in photocurrent as the carrier solely consists of photothermal-excited electrons without generating electron–hole pairs by electronic transition.^36^ Furthermore, the further increased density of SWCNTs also introduces the risk of recombination of photogenerated electron–hole pairs before reaching the electrodes, resulting in a consequential reduction in the carrier lifetime. In all, we proposed and optimized the delicate balance required for SWCNT density and channel length to enhance photodetector performance. The all-CNT photodetector with a density of #3 and channel length of 15 μm exhibits a high responsivity of 44 AW^–1^ under 532 nm laser (Figure 4e). In addition, the device exhibits good transient response, with a response time of around 4 ms (Figure 4f and Figure S8), comparable to the performance of previous CNT-based photoconductive detectors.^37−39^ High responsivity is related to the photoconductive gain (*G*), representing the number of carriers collected for each photogenerated carrier.^36,40^ Generally, the gain is determined by the ratio of τ_lifetime_ to the carrier transit time (τ_transit_). For our devices, the τ_lifetime_ was estimated to be ∼4.3 ms using the fall time of the photodetector, which is equivalent to the time taken for photoinduced carriers to recombine.^36,40−42^ The τ_transit_ and *G* were estimated to be ∼4.8 μs and ∼1000, respectively. The detailed calculation is provided in the Supporting Information. The high gain can be attributed to gas-synthesized high-purity s-SWCNTs (no structural damage and chemical contamination) and optimized density in the channel, which prevents the quenching of excitons and photogenerated carriers, resulting in an increased τ_lifetime_.^36^ By further improving the purity of the synthesized s-SWCNTs and shortening the channel length of the wearable device, the responsivity of the all-CNT photodetector is expected to be further improved.

Noise equivalent power (NEP) and specific detectivity (D*) are another two photoelectric performance metrics used to assess the sensitivity of photodetector to weak light signals.^37,43,44^ To accurately evaluate the sensitivity of our photodetector, we performed noise analysis on the dark current of the photodetector, and the measurement results are shown in Figure S9a. Under 0 V bias, white noise reaches 2 × 10^–13^ A/Hz^1/2^ and dominates most of the span of the measured frequency (0.01–25 Hz). However, at 1 V bias, the noise spectrum shows an evident 1/*f* characteristic with an exponent of 1.2 if fitted with , which is consistent with the trend observed previously in SWCNT devices.^36,38^ Accordingly, our all-CNT photodetector shows the NEP value ranging from 10^–10^ to 10^–12^ W/Hz^1/2^ at 1 Hz (Figure S9b) and is expected to exhibit lower NEP levels as 1/*f* noise is attenuated at high frequencies. In addition, we evaluated the detectivity of devices with different channel lengths and SWCNT densities, and the device with the highest responsivity has a detectivity of 1.9 × 10^9^ Jones (Figure S10).

Furthermore, delving deeper into the versatile characteristics of all-CNT photodetectors, their spectral absorption properties span a wide range, showcasing outstanding broadband absorption capabilities. This attribute positions them as up-and-coming candidates for achieving a broadband photoresponse. Employing the optimized photodetector as a foundation, we checked the photoresponse under varying light sources, including wavelengths of 532, 730, 900, and 1550 nm (Figure 4g). The outcomes reveal a broadband photoresponse within the visible and near-infrared light spectra. Especially noteworthy is the outstanding performance at a wavelength of 1550 nm, where the photoresponsivity exceeds 2 AW^–1^, and the detectivity also reaches 8.8 × 10^7^ Jones (Figure S11). The varied diameter^7,22^ (or band gap) of the high-quality s-SWCNTs in the channel accounts for the superior broadband photoresponse (Figure S12). Meanwhile, the photoresponse gradually diminishes as the wavelength (energy) of light increases (decreases), aligning with the findings reported in the literature.^45^Figure 4h and Table S1 comprehensively compare the key parameters and performance between our all-CNT photodetectors and previously reported CNT-based counterparts.^27,38,45−50^ Our photodetectors have a clear advantage in responsivity and comparable detectivity, although previously reported counterparts involve photolithography techniques and sorted high-purity s-SWCNTs (99%). It should be noted that some reported CNT-based devices^27,48^ show high detectivity, possibly due to low noise levels (assumed to be dominated by thermal and/or shot noise). However, these assumptions may not accurately describe the actual noise level of the devices. This exceptional achievement is credited to implementing a chemical-free method in the fabrication process, ensuring an absence of chemical contamination, and preserving the intrinsic properties of SWCNTs.

## Conclusions

In this study, we have introduced a scalable dry method that is lithography-free to fabricate wearable all-CNT device arrays based on FCCVD. Thanks to the integration of laser-assisted patterning and dry deposition methods, we have successfully fabricated wafer-scale device arrays with excellent uniformity and versatility. The device array exhibited favorable ohmic contact features, with minimal resistance fluctuations observed after bending, stretching, and humidity tests. This reveals their wearability and stability in diverse environmental conditions. Our approach addresses a few challenges in wearable devices associated with the fragility of rigid electrodes and the potential chemical contamination of materials. Moreover, the fabricated photodetectors demonstrate strong absorption capabilities ranging across wavelengths from visible to infrared, with a high responsivity of 44 AW^–1^ and a detectivity of 1.9 × 10^9^ Jones in the visible band, as well as a responsivity of 2 AW^–1^ and a detectivity of 8.8 × 10^7^ Jones at telecommunication wavelength. This study proposes an efficient, versatile, and scalable strategy for fabricating all-carbon nanotube device arrays. Significantly, this method is not limited to CNT materials but applies to flexible devices utilizing other gas-phase materials. This versatility holds promise for diverse applications in optoelectronics and wearable electronics.

## Methods

### Synthesis of DWCNTs

Flexible electrodes based on DWCNTs were prepared by FCCVD in the gas phase following our previous work.^25^ Specifically, ferrocene and thiophene were loaded into the high-temperature reactor through 300 sccm nitrogen (N_2_), and a flow of methane (CH_4_) and hydrogen (H_2_) was introduced simultaneously to synthesize DWCNTs at 1100 °C. DWCNTs are collected with a customized filter (thickness of 150 μm, with a pore size of 0.45 μm, Merck Millipore) to obtain a patterned electrode, which can be transferred to the target substrate using a dry-press method.^24^

### Synthesis of SWCNTs

A certain amount of ferrocene and thiophene was dissolved in isopropanol to form a precursor solution, which was then fed into the heating line at 130 °C through a syringe pump at a controlled rate of 1.0 μL min^–1^.^7^ Isopropanol, ferrocene, and thiophene served as carbon sources, catalysts, and promoters, respectively. Following the evaporation of the precursor solution, it was introduced into the FCCVD reactor at 880 °C under the carrier gases of 300 sccm N_2_ and 60 sccm H_2_. Comprehensive details on the synthesis process of SWCNTs can be found in our earlier report.^7^ Subsequently, as-synthesized SWCNTs were deposited on the top of patterned DWCNT electrode arrays using the direct thermophoretic method.^24^

### Fabrication of CNT transistors

The transistor structure based on both SWCNTs and DWCNTs remains consistent. The fabrication involves photolithography, metal deposition, and etching techniques, with standard channel length and width set to 100 μm. A 100 nm-thick SiO_2_ layer is the dielectric, p-type Si is the bottom gate, and Ti/Pd (5 nm/45 nm) is the source and drain electrode.

### Optical Measurement

The CNT films were transferred onto a substrate by the press-transferred method. Then, a UV–vis–NIR spectrometer (Agilent Cary 5000) was used to obtain optical absorption and transmission spectra. The optical absorption spectra were normalized by the π plasmon peak intensity. Furthermore, a Raman spectrometer (Horiba Jobin-Yvon Labram HR 800), equipped with an excitation wavelength of 633 nm, was used to acquire the Raman spectra of CNT films. Each sample was sampled at three random positions and averaged to avoid regional variability of samples. Raman spectra were finally normalized by graphite (G) band peak intensity.

### Microscopy Measurement

The optical photographs of the device were taken with a Leica DM6 optical microscope. The surface morphology and the density of CNT were characterized using an SEM (Zeiss Sigma VP) operated at 1 kV. For SEM imaging, CNTs with the same collection time used for devices were deposited on Si substrate to avoid the charging effect, especially when the density is low. Besides, a high-resolution TEM study of the microstructures of CNTs was carried out on a JEM 2200FS (JEOL Ltd.) double Cs-corrected electron microscope operated at an accelerating voltage of 200 kV. To know the thickness of DWCNT film, an AFM (Bruker) operated in a tapping mode was used to acquire topological images, which were then analyzed with Gwyddion software.

### Wearability and Environmental Stability Testing

The bending and stretching properties of all-CNT devices (on a PC substrate) were measured using a mechanical testing apparatus (Instron 5567, USA). The 400-cycle durability test was performed at a bending radius of 4.6 mm and a uniaxial rate of 0.5 mm min^–1^. A PDMS flexible substrate was used in the stretching test, and the uniaxial rate was set to 0.2 mm·min^–1^. The environmental stability test was conducted in a humidity-controlled chamber at room temperature. The humidity was jointly controlled through a humidifier and a dry nitrogen gas line. Before each formal test, the humidity was maintained for 1 h to allow the samples to stabilize fully.

### Electrical and Optoelectronic Measurements

The electrical characterization of bottom-gate transistors was performed using a PA200 probe station (equipped with a tungsten carbide needle, Suss Microtech) at room temperature and ambient conditions and a precision semiconductor parameter analyzer (Agilent 4156B) controlled simultaneously by a homemade LabVIEW program. In the photoelectric performance test, four power-adjustable lasers (532, 730, 980, and 1550 nm) were selected as light sources and illuminated on the photodetector through a 20× objective lens (NA = 0.4). The spot sizes of all lasers are smaller than the photodetector channel. The photodetectors were mounted on a sample holder (Linkam LN600-P) on the SNOM (WITec alpha300) platform. The *I–V* curves of the photodetectors were recorded using a homemade LabVIEW program by applying voltage using a source meter (Keithley 2401). To obtain transient photoresponse results, a chopper was employed to control the instantaneous light response of the device, and the data was read with an oscilloscope (Tektronix MDO3014). The noise measurements were performed with a setup^51^ under a dark environment and +1 V bias. The CNT sample, mounted in a PA200 probe station, was DC-voltage biased by an arbitrary waveform generator (Agilent Technologies 33510B). The current was amplified by a low-noise high-stability (LNHS) I to V converter (Basel Precision Instruments SP983c-IF), and its time trace of fluctuations was Fourier transformed using a FFT signal analyzer (Stanford Research Systems SR785). All these characterizations were performed at room temperature and atmospheric pressure.

## Supporting Information Available

Batch fabrication of all-CNT device arrays includes femtosecond laser processing schematics, optics images, SEM images, and *I–V* curves; current distribution in the device array; AFM image and height analysis of DWCNT electrode; optical and SEM images of DWCNT electrode after bending tests; calculation of the responsivity, photoconductive gain, noise equivalent power, and detectivity of all-CNT devices; rectification ratio and responsivity versus bias voltage of all-CNT photodetectors; responsivity as a function of incident power; current as a function of channel length and SWCNT density; transient photoresponse of all-CNT devices; noise analysis of all-CNT devices; detectivity as a function of channel length and SWCNT density under 532 nm laser; broadband response of all-CNT devices; diameter distribution of SWCNTs; relationship between incident photon energy and absorbance; performance comparison of the photodetectors using SWCNTs as the photoactive material (PDF)
