# Supplementary material for: Wafer-Scale Fabrication of Wearable All-Carbon Nanotube Photodetector Arrays
Source: ACS Nano. 2024 Jul 12;18(29):18900–9. doi: 10.1021/acsnano.4c01087 (PMC11271656; doi:10.1021/acsnano.4c01087)
Supplement: Supplementary file 1 — nn4c01087_si_001.pdf [file nn4c01087_si_001.pdf]

Supporting Information

Wafer-Scale Fabrication of Wearable All-Carbon Nanotube Photodetector Arrays

*Peng Liu[1,2], Er-Xiong Ding[2, *], Zhenyu Xu[1], Xiaoqi Cui[2], Mingde Du[2], Weijun Zeng[1,3], Anastasios Karakassides[1], Jin Zhang[2], Qiang Zhang[1, *], Faisal Ahmed[2], Hua Jiang[1], Pertti Hakonen[1,3], Harri Lipsanen[2], Zhipei Sun[2,3, *] and Esko I. Kauppinen[1, *]*

[1] Department of Applied Physics, Aalto University, Espoo, FI-00076, Finland.

[2] Department of Electronics and Nanoengineering, Aalto University, Espoo, FI-00076, Finland.

[3] QTF Centre of Excellence, Department of Applied Physics, Aalto University, Espoo, FI-00076, Finland

*E-mail: erxiong.ding@aalto.fi
*E-mail: qiang.zhang@aalto.fi
*E-mail: zhipei.sun@aalto.fi
*E-mail: esko.kauppinen@aalto.fi

1. Batch fabrication of all-CNT device arrays

For device fabrication, an Azpect/Newport laser micromachining system was utilized, featuring a Spectra-Physics Spirit 1040-16-SHG laser source with specifications including a 520 nm wavelength, 324 fs pulse duration, and 417 kHz repetition rate. This setup enabled the fabrication of devices with a resolution of approximately 10 μm (**Figure S1a**). A polyethylene terephthalate (PET) shadow mask with a customized pattern was created using femtosecond laser processing (10 minutes for a wafer-scale mask). It was subsequently paired with an MF-Millipore membrane filter through lamination (1 minute for the lamination process) (**Figure S1b-c**). The assembled filter with the pattern was placed at the outlet of the floating catalyst chemical vapor deposition (FCCVD) reactor to collect DWCNT film, with the thickness being precisely controlled by the collection time ranging from 5 to 30 minutes (**Figure S1d**). The collected DWCNT film on the patterned filter was transferred onto the target substrate by dry-press (< 1-minute transfer). The resulting structure on the transparent polycarbonate (PC) substrate is visually demonstrated in **Figure S1e**, while the optical microscope image (**Figure S1f**) and scanning electron microscope (SEM) image (**Figure S1g**) highlight the uniform and densely interconnected network of DWCNT patterns. Notably, electrical testing of the patterns reveals exceptional performance, showcasing an impressively low resistance of only 200 Ω (**Figure S1h**). Subsequently, a femtosecond laser was employed for secondary processing on the patterned DWCNTs to create a micrometer-level channel (10 minutes for a wafer-scale device array). **Figure S1i** displays a channel with a width of approximately 15 μm, and electrical tests were carried out on both sides of the channel to ensure successful separation and an insulating state (**Figure S1j**). This establishes the facile fabrication of micrometer-level channels, providing scalability and rapid manufacturing of flexible electronics. Finally, the laser-cut device arrays were placed in a cavity with a temperature gradient, and the channel material of as-synthesized SWCNTs were randomly deposited on the top of the device arrays based on the thermophoresis method we previously reported [1] (**Figure S1k**). The density of the channel material could be controlled by adjusting the temperature gradient, and deposition time ranges from 10 to 60 minutes. A typical SEM image of the material in the channel area is displayed in **Figure S1l**. As presented in **Figure S1m**, the two ends of the channel are re-conducted after depositing the channel material.

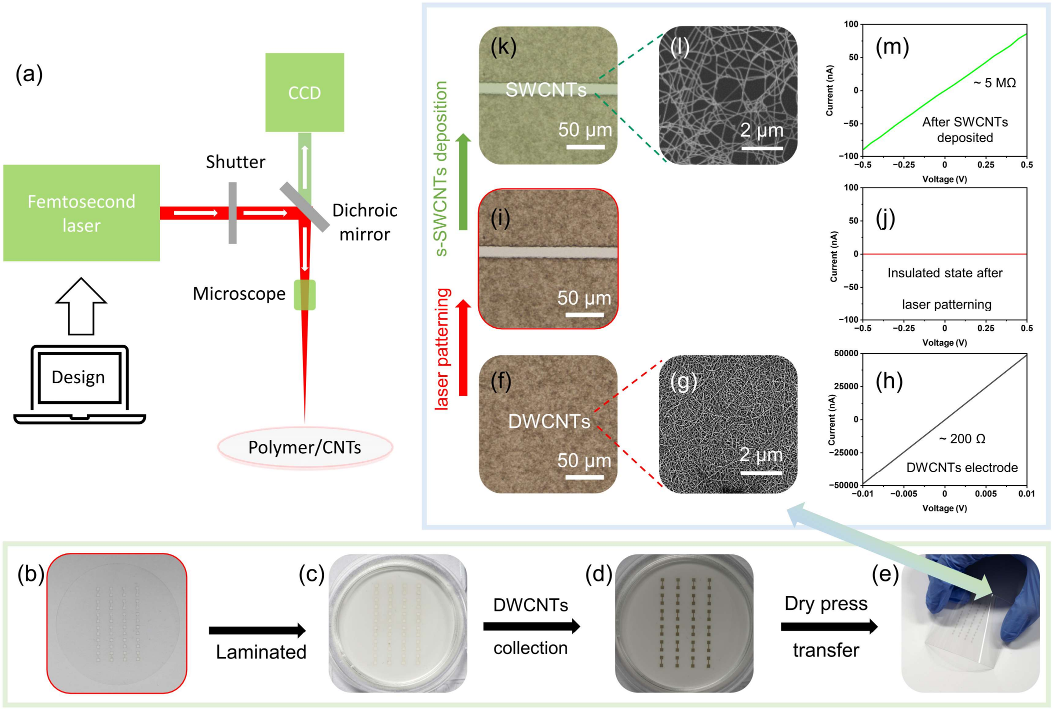

Figure S1. Schematics of femtosecond laser processing of all-carbon nanotube arrays. (a) Schematics of femtosecond laser processing. Optical images of (b) polymer masks after laser patterning, (c) patterned filter, (d) DWCNT array collected on a patterned filter, (e) device array transferred on a PC substrate. Optical images of DWCNT electrode (f) and channel by laser cutting (i) and device with SWCNT deposited (k), respectively. SEM images of DWCNTs in the electrode (g) and SWCNTs in the channel (l), respectively. *I-V* curve of the DWCNT electrode (h), patterned channel (j), and the device with SWCNTs deposited (m), respectively. The distance between the probes is about 2 mm for each *I-V* curve measurement.

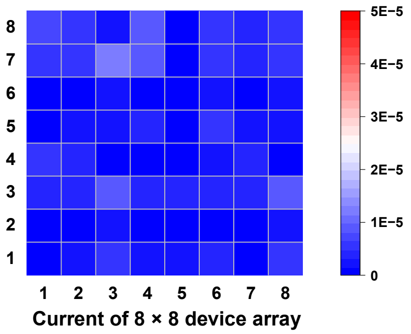

Figure S2. Distribution of current in an 8 × 8 device array.

2. Investigation of thickness and bending stability of the DWCNT electrode

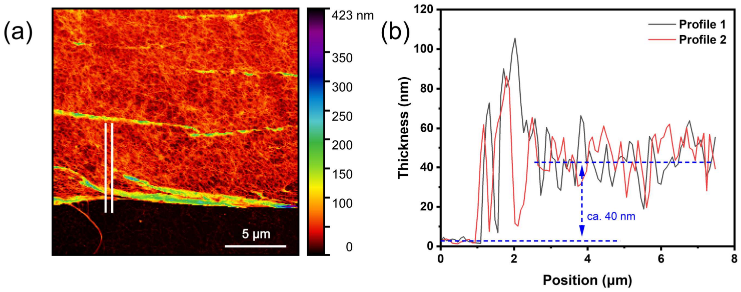

Figure S3. (a) AFM image of the edge of DWCNT electrode on a silicon substrate. The film was wetted with ethanol and dried before scanning. (b) Hight profiles of the DWCNT electrode edge with a thickness of approximately 40 nm.

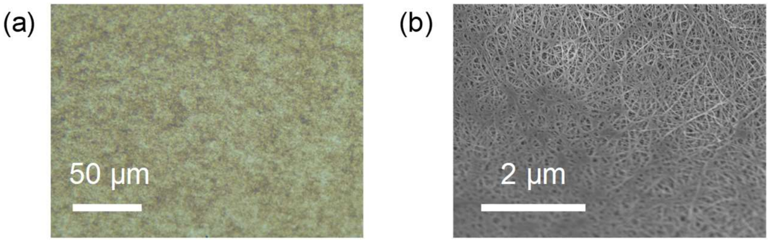

Figure S4. (a) Optical and (b) SEM images of the DWCNT electrode after over 400 bending cycles at a radius of 4.6 mm. No visible detachment of the DWCNT electrode was observed after bending. This robust mechanical performance is attributed to the flexibility of DWCNTs and the van der Waals contacts within the interconnected network structure.

3. Evaluation of optoelectronic performance

Responsivity (R_λ): A performance metric that evaluates the ability of a photodetector to convert an optical signal into an electrical signal, defined as the ratio of the output photocurrent (I_{ph}) to the input optical power (P), and the unit is A/W. The calculation of responsivity refers to our previous report,[2] as follows

$$R_\lambda = \frac{I_{\text{ph}}}{P} = \frac{I_{\text{light}} - I_{\text{dark}}}{P} \qquad (1)$$

where I_{light} and I_{dark} represent the current under illumination and dark environment, respectively.

Photoconductive gain(G): An essential parameter for evaluating the performance of the photodetector, and it is commonly determined by the ratio of carrier recombination lifetime ($\tau_{\ lifetime}$) to the carrier transit time ($\tau_{\ transit}$),[3,4] expressed as

$$G = \frac{\tau_{\ lifetime}}{\tau_{\ transit}} \qquad (2)$$

$$\tau_{\ transit} = \frac{L^2}{\mu V} \qquad (3)$$

In our devices[5], the holes are the major charge carriers, and the carrier's mobility ranges from 3.5 to 29.5 $cm^2\ V^{-1}\ s^{-1}$, with an average mobility of approximately 9.3 $cm^2\ V^{-1}\ s^{-1}$. Based on the formula (3), where L is the channel length of 15 μm, μ is the mean hole mobility of 9.3 $cm^2\ V^{-1}\ s^{-1}$, and V is the bias, the $\tau_{\ transit}$ is estimated to be about 4.8 μs. In addition, the τ_{lifetime} was estimated to be 4.3 ms using the fall time of the photodetector, which is equivalent to the time taken for photoinduced carriers to recombine.[4,6–8] According to the formula (2), the photoconductive gain was estimated as 1000.

Response time: A crucial indicator for evaluating photodetector performance, often characterized by rise time and fall time. Rise time is typically defined as the time taken for the photocurrent to switch from 10% to 90% of the maximum photocurrent and vice versa as the fall time.

Noise equivalent power (NEP) and detectivity (D^*): NEP characterizes the minimum optical signal power that can be distinguished from the photodetector noise. It is defined as the incident optical power value of the device when the signal-to-noise ratio is equal to 1, and the unit is $W/Hz^{1/2}$. However, it is difficult to compare the performance of different photodetectors with different structures and mechanisms by NEP. Therefore D^* was employed to widely

compare the ability of photodetector to detect weak light signals. It represents the signal-to-noise ratio produced per unit area of photodetector under unit incident power, and the unit is Jones or cm·Hz$^{1/2}$·W^{-1}. The calculation formulas are as follows.[9–11]

$$NEP = \frac{I_{Noise}}{R_{\lambda}} \qquad (4)$$

$$D^* = \frac{\sqrt{A}}{NEP} \qquad (5)$$

where I_{Noise} and A represent noise equivalent power and channel area of the photodetector, respectively.

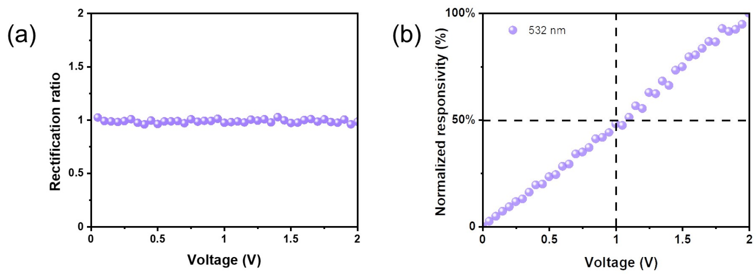

Figure S5. (a) Rectification ratio and (b) Responsivity *versus* bias voltage of all-CNT photodetectors.

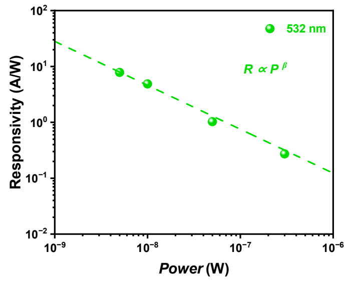

Figure S6. Responsivity as a function of incident power under 532 nm laser.

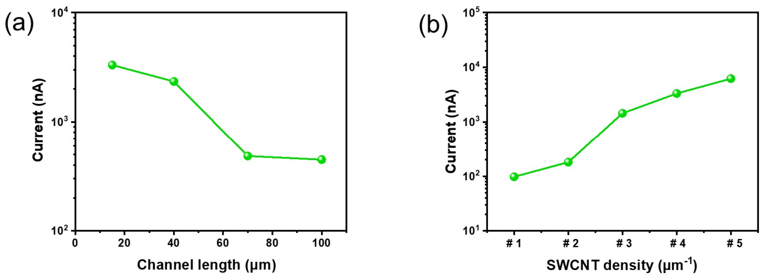

Figure S7. Current is a function of (a) channel length and (b) SWCNT density under a laser of 532 nm.

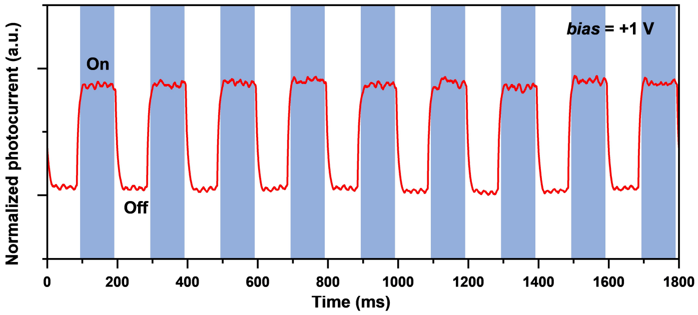

Figure S8. Transient photoresponse of all-CNT devices. Time-dependent photocurrent was measured under 1 V bias, 532 nm laser with a switched On/Off frequency of 10 Hz, implemented with an optical chopper and an oscilloscope.

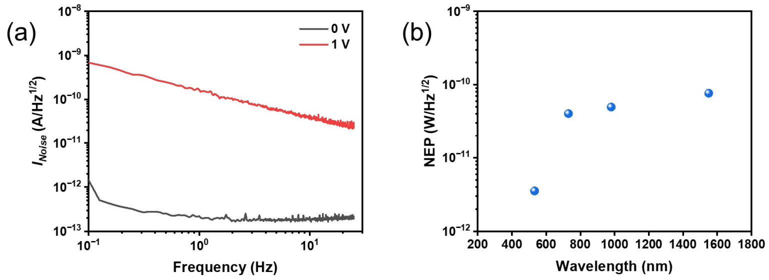

Figure S9. (a) Noise spectral density as a function of frequency. The device was operated at room temperature, ambient, and dark environments, with voltages of 0 V and 1 V. (b) Noise equivalent power of an all-CNT photodetector.

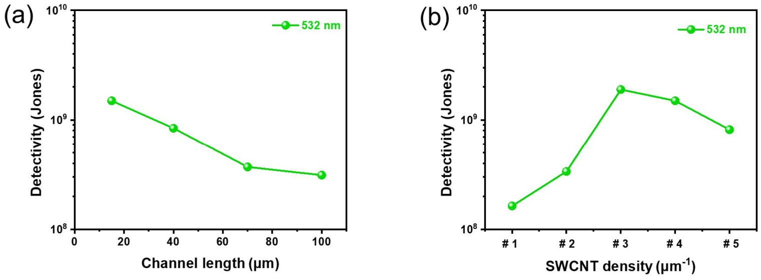

Figure S10. Detectivity as a function of (a) channel length and (b) SWCNT density under 532 nm laser.

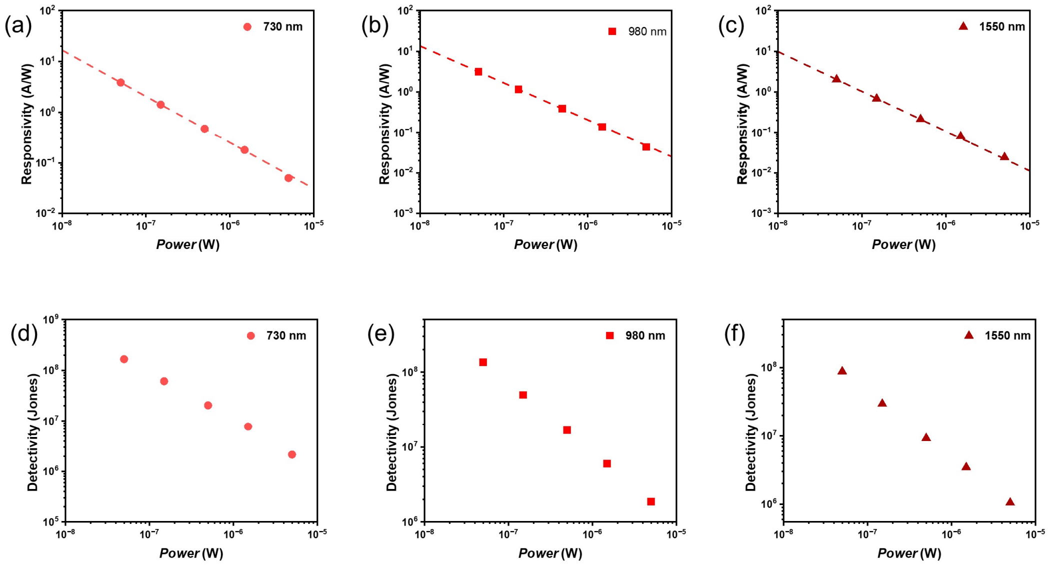

Figure S11. (a, b, c) Responsivity and (d, e, f) Detectivity of all-CNT photodetector under lasers of 730 nm, 980 nm, and 1550 nm, respectively.

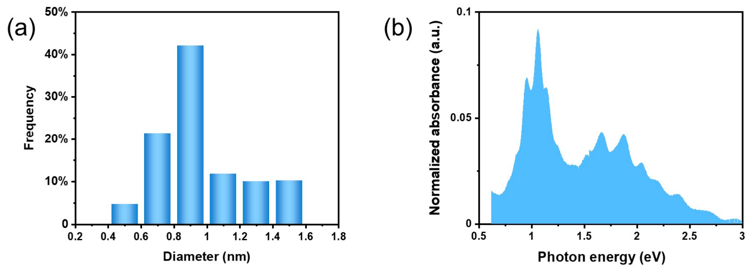

Figure S12. (a) Fitted diameter distributions of the SWCNTs from the optical absorption spectrum in Figure 2d. This result was obtained by fitting the optical absorption spectrum with a MATLAB code.[5,14] (b) The relationship between incident photon energy and absorbance.

Table S1. Performance comparison of the photodetectors using SWCNTs as the photoactive material.

Fabrication	Electrode	Channel materials	Voltage (V)	Wavelength (nm)	Responsivity (A/W)	Detectivity (Jones)	Wearable	Scalability	Reference
Lithography + Wet transfer	Graphene	Sorted s-SWCNTs (~99%)	1	524	0.023	3.70×10^{9} @	No	No	**Ref. 27**
Lithography + Wet transfer	Au	Sorted s-SWCNTs	0.5	532	0.293	N/A	No	No	**Ref. 47**
Lithography + Wet transfer	Au	Sorted SWCNTs (6, 5)-enriched	1	1064	727.0	2.97×10^{11} @	No	No	**Ref. 48**
Lithography + Wet transfer	Au	Sorted s-SWCNTs (~98%)	10	1150	0.016	2.30×10^{8}	No	No	**Ref. 38**
Lithography + Wet transfer	Hf + Pd	Sorted s-SWCNTs (> 99.9999%)	1.5	1550	1.500	2.24×10^{8}	No	No	**Ref. 46**
Lithography + Wet transfer	Hf + Pd	Sorted s-SWCNTs (> 99.9999%)	0.5	2000	0.620	N/A	No	Yes	**Ref. 49**
Lithography + Dry transfer	Au	As-synthesized SWCNTs	0.2	700	0.065	N/A	No	No	**Ref. 50**
Lithography + Dry transfer	Au	As-synthesized SWCNTs	0.2	700	0.026	1.35×10^{8} @	No	No	**Ref. 45**
				1100	0.023	1.20×10^{8} @			
				1550	0.017	8.00×10^{7} @			
				2000	0.014	7.13×10^{7} @			
**Laser patterning + Dry transfer**	DWCNTs	As-synthesized SWCNTs	1	532	44.04	1.90×10^{9}	Yes	Yes	**This Work**
				730	3.857	1.66×10^{8}			
				980	3.136	1.35×10^{8}			
				1550	2.034	8.76×10^{7}			

@ It is assumed that the noise is dominated by shot noise, and the detectivity calculation is based on the formula $D^{*} = RA^{1/2}(2qI_{dark})^{-1/2}$, where R, A, q, and I_{dark} represent the responsivity, channel area, elementary charge, and dark current of the photodetector, respectively.

References

(1) Laiho, P.; Mustonen, K.; Ohno, Y.; Maruyama, S.; Kauppinen, E. I. Dry and Direct Deposition of Aerosol-Synthesized Single-Walled Carbon Nanotubes by Thermophoresis. *ACS Appl. Mater. Interfaces* **2017**, *9*, 20738–20747.

(2) Ding, E. X.; Liu, P.; Yoon, H. H.; Ahmed, F.; Du, M.; Shafi, A. M.; Mehmood, N.; Kauppinen, E. I.; Sun, Z.; Lipsanen, H. Highly Sensitive MoS_2 Photodetectors Enabled with a Dry-Transferred Transparent Carbon Nanotube Electrode. *ACS Appl. Mater. Interfaces* **2022**, *15*, 4216–4225.

(3) Wu, W.; Yang, F.; Fang, X.; Cai, X.; Liu, X.; Zhang, F.; Wang, S. Ultrafast Carbon Nanotube Photodetectors with Bandwidth over 60 GHz. *ACS Photonics* **2023**, *10*, 1060.

(4) Park, S.; Kim, S. J.; Nam, J. H.; Pitner, G.; Lee, T. H.; Ayzner, A. L.; Wang, H.; Fong, S. W.; Vosgueritchian, M.; Park, Y. J.; Brongersma, M. L.; Bao, Z. Significant Enhancement of Infrared Photodetector Sensitivity Using a Semiconducting Single-Walled Carbon Nanotube/C_{60} Phototransistor. *Adv. Mater.* **2015**, *27*, 759–765.

(5) Liu, P.; Khan, A. T.; Ding, E.; Zhang, Q.; Xu, Z.; Bai, X.; Wei, N.; Tian, Y.; Li, D.; Jiang, H.; Lipsanen, H.; Sun, Z.; Kauppinen, E. I. Direct Synthesis of Semiconducting Single-Walled Carbon Nanotubes Toward High-Performance Electronics. *Adv. Electron. Mater.* **2023**, 2300196.

(6) Konstantatos, G.; Badioli, M.; Gaudreau, L.; Osmond, J.; Bernechea, M.; de Arquer, F. P. G.; Gatti, F.; Koppens, F. H. L. Hybrid Graphene–Quantum Dot Phototransistors with Ultrahigh Gain. *Nat. Nanotechnol.* **2012**, *7*, 363–368.

(7) Cai, B.; Su, Y.; Tao, Z.; Hu, J.; Zou, C.; Yang, Z.; Zhang, Y. Highly Sensitive Broadband Single-Walled Carbon Nanotube Photodetectors Enhanced by Separated Graphene Nanosheets. *Adv. Opt. Mater.* **2018**, *6*, 1800791.

(8) Liu, Y.; Wang, F.; Wang, X.; Wang, X.; Flahaut, E.; Liu, X.; Li, Y.; Wang, X.; Xu, Y.; Shi, Y.; Zhang, R. Planar Carbon Nanotube-Graphene Hybrid Films for High-Performance Broadband Photodetectors. *Nat. Commun.* **2015**, *6*, 8589.

(9) Fang, Y.; Armin, A.; Meredith, P.; Huang, J. Accurate Characterization of Next-Generation Thin-Film Photodetectors. *Nat. Photonics* **2019**, *13*, 1–4.

(10) Cai, X.; Wang, S.; Peng, L.-M. Recent Progress of Photodetector Based on Carbon Nanotube Film and Application in Optoelectronic Integration. *Nano Res. Energy* **2023**, *2*, e9120058.

(11) Wang, F.; Zhang, T.; Xie, R.; Wang, Z.; Hu, W. How to Characterize Figures of Merit of Two-Dimensional Photodetectors. *Nat. Commun.* **2023**, *14*, 2224.

(12) Lu, R.; Christianson, C.; Kirkeminde, A.; Ren, S.; Wu, J. Extraordinary Photocurrent Harvesting at Type-II Heterojunction Interfaces: Toward High Detectivity Carbon Nanotube Infrared Detectors. *Nano Lett.* **2012**, *12*, 6244–6249.

(13) Liu, Y.; Yin, J.; Wang, P.; Hu, Q.; Wang, Y.; Xie, Y.; Zhao, Z.; Dong, Z.; Zhu, J. L.; Chu, W.; Yang, N.; Wei, J.; Ma, W.; Sun, J. L. High-Performance, Ultra-Broadband, Ultraviolet to Terahertz Photodetectors Based on Suspended Carbon Nanotube Films. *ACS Appl. Mater. Interfaces* **2018**, *10*, 36304–36311.

(14) Ding, E.-X.; Liu, P.; Khan, A. T.; Zhang, Q.; Wei, N.; Jiang, H.; Kauppinen, E. I. Towards the Synthesis of Semiconducting Single-Walled Carbon Nanotubes by Floating-Catalyst Chemical Vapor Deposition: Challenges of Reproducibility. *Carbon* **2022**, *195*, 92–100.
